# Hydatid Cysts of Liver

**Published:** 1890-12-27

**Authors:** 


### HYDATID CYSTS OF LIVER.

Krause, the pupil of Volkmann, and one of the most prominent of the younger surgeons in Germany, utters a word of warning against exploratory punctures of these cysts, suggested by a case of peculiar interest that had come under his notice. He recommends that such punctures should be made with the finest needle possible, and that every preparation should be made for proceeding with the operation of complete evacuation and incision in the event of echinococci being detected in the fluid, so as to avoid the danger of their escaping into the cavity of the peritoneum. The case was that of a man who had noticed a tumour of the liver three years previously, when an exploratory puncture was made, but with what results was not known. Six months later the abdomen began to swell, and in eighteen months more the right half was felt to be full of globular tumours. Krause performed laparotomy which revealed an enormous number of echinococcus cysts enveloped in a common fibrous sac or covering membrane. One larger than the rest was firmly adherent to the under surface of the liver, the others varied in size, from that of a hazel-nut to that of an orange, and all contained innumerable daughter cysts. The operation of removing these had been conducted for two and a half hours, when it was stopped in consequence of impending collapse, and a few of the cysts were unavoidably left behind. The patient recovered, but will probably have to undergo a repetition of the operation. Krause has no doubt as to this condition having resulted from the escape of the fluid containing echinococci into the peritoneum, when the original sac was punctured, a contingency which should always be borne in mind.

